# Metabolomics analysis of salvage chemotherapy on refractory acute myeloid leukemia patients†

**DOI:** 10.1039/c7ra13298k

**Published:** 2018-04-18

**Authors:** Zhi Zheng, Pingyi Liu, Liting Xu, Zhiqiang Peng, Yayue Zhang, Xinyi Chen, Li Hou, Wenhao Cui, Fangfang Tou, Jun Rao, Xing Fan

**Affiliations:** Jiangxi Cancer Hospital, Jiangxi Cancer Center, Medical College of Nanchang University Nanchang 330029 P. R. China raojun1986@126.com; Laboratory of Department of Hematology, Yishui Central Hospital of Linyi Linyi Shandong Province 276400 P. R.China; Department of Hematology and Oncology, Beijing University of Chinese Medicine Beijing 100029 P. R.China; Departments of Pharmacology, Kyoto Prefectural University of Medicine Kyoto 6028566 Japan; Department of Hematology, Shanghai Institute of Hematology, Ruijin Hospital, Shanghai Jiaotong University School of Medicine Shanghai 200025 P. R. China fx-86@163.com

## Abstract

Acute myeloid leukemia (AML) is a group of hematological malignancies causing high mortality around the world. However, the treatment of AML is still one of the most formidable challenges. In this study, we employed a well-established global metabolic profiling platform, which combined ultra-performance liquid chromatography-tandem mass spectrometry (UPLC-MS/MS) with gas chromatography mass spectrometry (GC-MS) to investigate the metabolic alterations associated with salvage chemotherapy on 10 refractory acute myeloid leukemia (RAML) patients. A total of 390 metabolites were identified from 20 serum samples obtained from all 10 patients before and post salvage chemotherapy. The metabolomics profile was found to be very heterogeneous across the RAML patients. The results showed very subtle metabolic differences upon one-time chemotherapy treatment for an individual patient. Only 9 metabolites including imidazole lactate, glycerol 3-phosphate, three fatty acids, and four lysolipids in the blood serum were significantly changed before and post chemotherapy, suggesting their important roles during the development of RAML. This study may not only provide new insight into the metabolomics features in RAML patients, but also have relevance to improve the treatment and outcome of RAML.

## Abstract 2

Salvage chemotherapy had minimal impact on the metabolomics for individual RAML patient.

## Graphical abstract

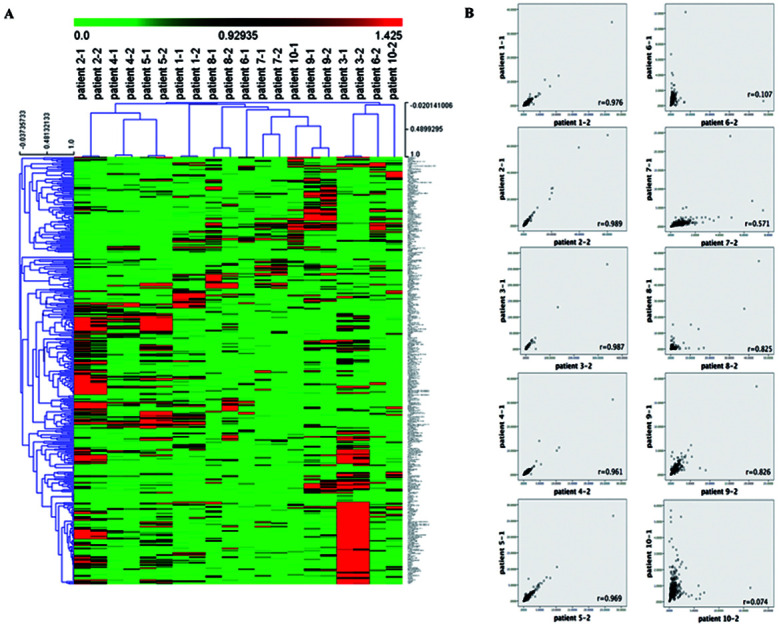

## Introduction

Acute myeloid leukemia (AML) is the most common acute leukemia in adults, which is characterized by the accumulation of immature myeloid precursors, and escape apoptosis, ultimately resulting in the inhibition of normal hematopoiesis. Over the past few decades, the outlook for patients with AML has been improved due to refinements in the diagnosis and therapy.^1^ However, the prospects for AML patients remains very dismal, especially for refractory acute myeloid leukemia (RAML). RAML is a group of hematological malignancies with high heterogeneity, which could not be simply classified by the cytogenetic/cytomolecular or immunophenotype. Although, many new chemotherapeutic and nonchemotherapeutic agents have been used for treating RAML, patients are less likely to respond to any treatment with estimated overall survival (OS) at no more than 10% at 3 years.^2–5^

Over the past few years, a great number of genomic studies have identified a series of genes that are affected by certain recurrent somatic point mutations in various AML subtypes, which led to a better understanding on the molecular mechanism of AML development and suggested new therapeutic strategies for the disease.^6^ For example, Chen *et al.* found that an about 50% reduction in gene dosage in the mixed lineage leukemia 3 (MLL3) gene (located on 7q36.1) rather than complete loss, cooperated with other events occurring in -7/del(7q) AMLs, resulting in the development of leukemogenesis.^7^ The mouse model functionally identified MLL3 as a haplo insufficient 7q tumor suppressor and meanwhile suggested a potential therapeutic option for the aggressive disease. Moreover, the Cancer Genome Atlas Research Network reported the results of genomes from 200 AML patients and defined 11 genes were mutated in AML with different functional categories.^8^ Other genomic studies revealed that gene mutations in DNMT3A, ASXL1, and TET2 play essential roles in clonal expansion of pre-leukemic hematopoietic stem cells, and might be related with the relapse.^9–11^ These gene mutations are the primary therapeutic targets for developing new treatment regimens for AML and RAML.

Meanwhile, other innovated high throughput approaches including transcriptomics and proteomics are developed to study the mechanisms of AML and the disease clinical features at molecular levels. For example, Maiga *et al.* conducted transcriptome analysis of G protein-coupled receptors in distinct genetic subgroups of AML, and then identified different potential therapeutic targets.^6^ Perna *et al.* integrated large transcriptomics and proteomics datasets from both malignant and normal tissues, and developed an algorithm to identify potential targets expressed in leukemia stem cells instead of normal hematopoietic cells. The results finally identified several target pairings, holding great promise for systematic combinatorial chimeric antigen receptor therapy of AML.^12^ In addition, Visconte *et al.* performed comprehensive quantitative proteomic profiling of the pharmacodynamic changes induced by MLN4924 in MV4-11 FLT3 ITD+ acute myeloid leukemia cells.^13^ This study evaluated the global impact of inhibiting NEDDylation with MLN4924 on the AML proteome and established rationale for its combination with azacytidine to treat the tumor *in vivo*.

Recently, advanced metabolomic profiling methods have been proven to be a powerful tool to comprehensively and semi-quantitatively determine global metabolites in specific cells, tissues, or bodily fluids.^14^ More importantly, increasingly evidences of metabolomics' role in tumor diagnosis and therapy were appearing including metabolomics investigations on various cancers such as colorectal cancer (CRC), gastric cancer, pancreatic cancer, and liver cancer.^15–19^ The main analytical platforms for metabolomics analysis include nuclear magnetic resonance (NMR) spectroscopy and mass spectrometry (MS).^20,21^ Mass spectrometry-based metabolic methods including liquid chromatography mass spectrometry (LC-MS) or gas chromatography-mass spectrometry (GC-MS) are increasingly favored due to their unique advantages such as high sensitivity and wide range of molecules.^20,21^ Among them, UPLC-MS is especially considered as one of the best analytical techniques in animal model research and clinical studies including liver, lung, gastrointestinal, urogenital and other diseases.^22–27^ For example, Chen *et al.* (2017) recently collected 180 chronic kidney disease (CKD) patients and 120 age-matched healthy controls, and conducted metabolomic studies by utilizing UPLC-HDMS (Waters) and the gene profiling using quantitative real-time RT-PCR techniques.^25^ They showed correlations between identified metabolites and gene expression of inflammation/Wnt/β-catenin signaling cascade, which illuminated the molecular pathogenesis of patients with advanced CKD.

The metabolomics study on RAML may help to further explore of the intrinsic disease and expand the arsenal of effective therapeutically modalities for RAML patients. For instance, metabolomics study on leukemia cell line treated with chemotherapeutics *in vitro* has been reported in the Jurkat cell line as a model of acute lymphoblastic leukemia, reflecting the metabolomics data might provide the potential therapy biomarker for RAML patients in the future.^28^ Accordingly, in this study, we for the first time employed an established non-targeted metabolomic profiling platform that combined UPLC/MS/MS with GC/MS to measure the metabolic profiles in 10 RAML patients before and after salvage chemotherapy treatment. The results from this study may shed new light on RAML pathogenesis and treatment.

## Experimental

### Patients

From to January 2016 to December 2016, a total of 10 RAML patients from Jiangxi Cancer Hospital were included in this retrospective study. Informed consent was obtained from all patients, in accordance with the regulations of the Jiangxi Cancer Hospital Institutional Review Boards. Patients were numbered from 1 to 10.

### Diagnosis

Diseases diagnosis was established according to World Health Organization (WHO) classification.^29^ RAML was defined: primary induction failure (PIF) after 2 cycles of chemotherapy, first early relapse after a remission duration less than 6 months, relapse disease refractory to salvage chemotherapy containing high-dose Ara-C.^30^ Patients enrollment eligibility criteria listed below: ① meet the diagnostic criteria for RAML; ② no chemotherapy used at least for one month. The exclusion criteria were: ① combined with severe heart, brain, liver, kidney disease; ② mental illness; ③ glaucoma; ④ pregnancy, lactating women; ⑤ known to the drug allergy; ⑥ age less than 10 years old or elder than 80 years old.

### Samples collection and metabolomics analysis

About 5 mL serums from each RAML patient before (baseline) and post chemotherapy were collected for sample preparation. Before the baseline serum samples collection, the RAML patients were off chemotherapy for at least one month, and the post-chemotherapy serum was collected at 30 days after chemotherapy. Global serum metabolic profiles were then determined by a global unbiased platform which is a combination of three independent analytical platforms: UPLC/MS/MS optimized for basic species, UHLC/MS/MS optimized for acidic species, and GC/MS optimized for small, volatile, and thermally stable molecules.^31^ In this study, both the GC-MS platform (Thermo Ultra GC-ISQ, Waltham, MA, USA) and LC-MS platform (Waters ACQUITY UPLC Milford, MA, USA-Thermo LTQ XL, Milford, Waltham, MA, USA) were adopted, which has been widely applied to metabolomic studies.^32–36^

For GC/MS analysis, the samples were derivatized using bistrimethyl-silyl-triflouroacetamide (BSTFA, Sigma-Aldrich, St. Louis, MO, USA) prior to injection.^31^ For UPLC/MS/MS analysis, each sample was analyzed using two separate dedicated columns: one for negative ions and one optimized for positive ions. The mobile phase for negative ion analysis consisted of 6.5 mM ammonium bicarbonate (Sigma-Aldrich, St. Louis, MO, USA), pH 8.0 (solvent A) and 6.5 mM ammonium bicarbonate in methanol (Sigma-Aldrich, St. Louis, MO, US) (solvent B), while in positive ion mode, 0.1% formic acid (Sigma-Aldrich, St. Louis, MO, USA) in H_2_O (solvent A) and 0.1% formic acid in methanol (solvent B) were used. Furthermore, gradient was directly eluted into the mass spectrometer from 0% solvent B to 98% solvent B at a flow rate of 350 μL min^−1^ over 11 min. Additionally, the retention time, molecular weight (*m*/*z*), and tandem mass spectrometry (MS/MS2) spectra of all detectable ions for each sample were measured in MS analysis, which alternated between MS (99–1000 *m*/*z*) and data-dependent MS2 scans using dynamic exclusion. The type and content of all metabolites before (day 0, baseline) and after treatment (day 30, post-chemotherapy) in RAML patients were identified by automated comparison to Metabolon's reference library entries.^31^ The library has already been established using approximately 1500 authentic standards, which were analyzed in multiple concentrations and under the same conditions as the experimental samples. In total, 390 of metabolites were detected and analyzed from all the blood serum samples of the 10 RAML subjects. The differences of metabolomics between baseline samples and post-treatment samples were analyzed, especially the changes of amino acid metabolic pathway, carbohydrate metabolic pathway and lipid metabolism pathway.

### Statistical analysis

To normalize the metabolites for data analyses, a data normalization step was performed by registering the median level of each compound to equal to one (1.00). And meanwhile, the missing values (if any) were assumed to be below the limits of detection and were imputed with the observed minimum values. Log transformation of normalized data, ANOVA contrasts, Welch's two-sample *t*-test and paired *t*-test were used to identify biochemical which were significantly different between before and post chemotherapy in RAML patients.^31^*P* values less than 0.05 was defined as statistical significance. SPSS 17.0 (IBM, New York, US) and MultiExperiment Viewer 4.8 software packages were used for data analysis.^37^ The metabolic data was visualized by K-Medians clustering method and stoichiometry including principal component analysis (PCA) and partial least squares discriminant analysis (PLS-DA), and SPSS scatter dot plots. SIMCA-P software (v13.0, Umetrics, Malmö, Sweden) was used in this study.

## Results and discussion

### Clinical characteristics of RAML patients

The clinical characteristics of the 10 enrolled patients were summarized in Table 1. The median age of the patients was 58.5 year-old (range, 51–67 years), and 7 out of 10 patients were male. All patients had good performance status with the Eastern Cooperative Oncology Group (ECOG) less than 3 and the average of Karnofsky Performance Score (KPS) more than 70.7 of the 10 patients were diagnosed with *de novo* AML and 3 patients were secondary AML. 6 of 10 patients appeared normal karyotypes, however, the patient 4 had complex karyotype, patient 3 was 46, XY, 20q-, and the onset cytogenetic results of patient 5 and 9 are not available. 6 out of 10 patients have molecular alterations as shown in Table 1. At the baseline time point, 3 patients acquired complete remission (CR), patient 8 was partial remission (PR), 4 patients had no response (NR) to previous treatments, and patients 1 and 3 were at the first relapse (R1). The chemotherapy regimens applied to each patient were shown in the Table 1, include HD-Ara-c (high dose cytarabine, 1.5 g m^−2^ or 2.0 g m^−2^ q 12 h for over 3 days), CAG (cytarabine, aclarubicin, G-CSF), 2-CDA with CAG (Cladribine, cytarabine, aclarubicin, G-CSF) and HA (homoharringtonine and cytarabine). 30 days after these chemotherapy treatments, serum samples were collected for metabolomics analysis. After this chemotherapy, the 3 CR patients were still remaining CR, patient 8 showed progressive disease (PD), the previous 4 NR patients remained NR and the two patients at R1 showed NR for this chemotherapy. These results indicated that the one time chemotherapy did not change the disease status.

**Table 1:** Clinical features of the patients^a^

Patient	Sex, age (years)	Diagnosis	% blast in BM	Cytogenetics	Molecular alterations	Disease status before	Chemotherapy regimens	Disease status after
1	F, 65	*De novo* AML	47	Normal	*CEBPA* Insertion	R1	HD-Ara-c	NR
2	M, 67	Secondary AML	28	Normal	*WT1*, *MLL-PTD*, *PRAME*, *JAK2*	NR	HD-Ara-c	NR
3	F, 56	Secondary AML	34	20q-	*WT1*, *PRAME*,	R1	CAG	NR
4	M, 55	*De novo* AML	55	Complex	*NPM1*	NR	CAG	NR
5	M, 66	*De novo* AML	90	NA	*NPM1*	NR	2-CDA + CAG	NR
6	M, 51	Secondary AML	34	Normal	*WT1*, *MLL-PTD*, *PRAME*, *JAK2*	NR	HD-Ara-c	NR
7	M, 55	*De novo* AML	30	Normal	None	CR	HD-Ara-c	CR
8	M, 56	*De novo* AML	42	Normal	None	PR	HD-Ara-c	PD
9	F, 61	*De novo* AML	64	NA	None	CR	HA	CR
10	M, 62	*De novo* AML	38	Normal	None	CR	HD-Ara-c	CR

a F: female; M: male; R1: relapse 1; NR: no remission; CR: complete remission; PR: partial remission; PD: progressive disease; HD-Ara-c: high dose cytarabine; CAG: cytarabine + aclarubicin + G-CSF; 2-CDA: Cladribine; HA: Homoharringtonine and cytarabine.

### Clinical outcome revealed by peripheral leukocyte, erythrocyte and platelet count post the chemotherapy

We have monitored the hematological clinical outcomes before and after the chemotherapy along with the metabolomics analyses. As shown in ESI Table 1,† the white blood cells (WBC) count of the 10 RAML patients after chemotherapy on day 15 was significantly lower compared to the WBC count on day 0 at the baseline (4.119 ± 2.657 × 10^9^ L^−1^*vs.* 10.198 ± 9.836 × 10^9^ L^−1^, *p* = 0.048), indicating acute chemotherapy cytotoxicity effect on WBC. However, WBC number on day 30 had no significant difference compared to day 0 (5.467 ± 4.392 × 10^9^ L^−1^*vs.* 10.198 ± 9.836 × 10^9^ L^−1^, *p* = 0.076). The hemoglobin level was also decreased obviously on day 15 (71.0 ± 13.3g L^−1^*vs.* 80.8 ± 16.8 g L^−1^, *p* = 0.002) and recovered on day 30 (76.7 ± 17.6 g L^−1^*vs.* 80.8 ± 16.8 g L^−1^, *p* = 0.172). Similar occurred on the platelet count (day 15 *vs.* day 0: 60.0 ± 53.8 × 10^9^ L^−1^*vs.* 93.2 ± 88.3 × 10^9^ L^−1^, *p* = 0.042; day 30 *vs.* day 0: 87.1 ± 85.3 × 10^9^ L^−1^*vs.* 93.2 ± 88.3 × 10^9^ L^−1^, *p* = 0.234). The results indicated that the chemotherapy on the RAML patients resulted in significant cell cytotoxicity on hematopoietic cells.

### Treatment outcome revealed by biochemical examination

We did the biochemical examination on the 10 RAML patients before and post the chemotherapy (ESI Table 1†). The results showed no significant changes in serum biochemical indicators for liver function. The alanine aminotransferase (ALT) levels on day 15 and day 30 compared to day 0 were 21.5 ± 13.0 U L^−1^*vs.* 20.2 ± 8.9 U L^−1^, *p* = 0.577 and 23.6 ± 9.5 U L^−1^*vs.* 20.2 ± 8.9 U L^−1^, *p* = 0.222, while the aspartate transaminase (AST) levels were also had no significant changes (day 0 *vs.* day 15: 22.5 ± 11.7 U L^−1^*vs.* 20.1 ± 5.4 U L^−1^, *p* = 0.480; day 0 *vs.* day 30: 24.6 ± 9.9 U L^−1^*vs.* 20.2 ± 8.9 U L^−1^, *p* = 0.154). We did not observe significant changes of serum urea nitrogen (BUN) and creatinine (Ccr) on day 15 and day 30 of the RAML patients post chemotherapy (BUN day 15 *vs.* day 0: 7.81 ± 7.65 mg dL^−1^*vs.* 8.53 ± 9.01 mg dL^−1^, *p* = 0.809; BUN day 30 *vs.* day 0: 6.75 ± 4.36 mg dL^−1^*vs.* 8.5 3 ± 9.01 mg dL^−1^, *p* = 0.515; Ccr day 15 *vs.* day 0: 70.46 ± 38.89 μmol L^−1^*vs.* 79.82 ± 60.50 μmol L^−1^, *p* = 0.392; Ccr day 30 *vs.* day 0: 68.73 ± 37.65 μmol L^−1^*vs.* 79.82 ± 60.50 μmol L^−1^, *p* = 0.259) (ESI Table 1†). These results indicated that the liver and renal functions of these RAML patients were stable during this one-time chemotherapy.

### Metabolomics analysis of serum from RAML patients before and post chemotherapy

We have noticed this one-time chemotherapy had minimal or no effect on the renal function and liver function, therefore these organ functions should have limited affection to metabolomics *in vivo* in patients. We have performed the metabolomics analysis on the blood serum from the RAML patients that could potentially reveal the effect of the chemotherapy on metabolomics. In total, 390 metabolites (ESI Table 2†) of chemical structure were identified from all 10 subjects. These metabolites included 94 amino acids, 29 carbohydrates, 154 lipids, 17 nucleotides, 22 vitamins cofactors and vitamins, 16 peptides, 10 energy-related compounds, and 48 exogenous compounds. The 390 metabolites specifically involved in the 8 major metabolic pathways and 71 sub-metabolic pathways.

**Fig. 1:**
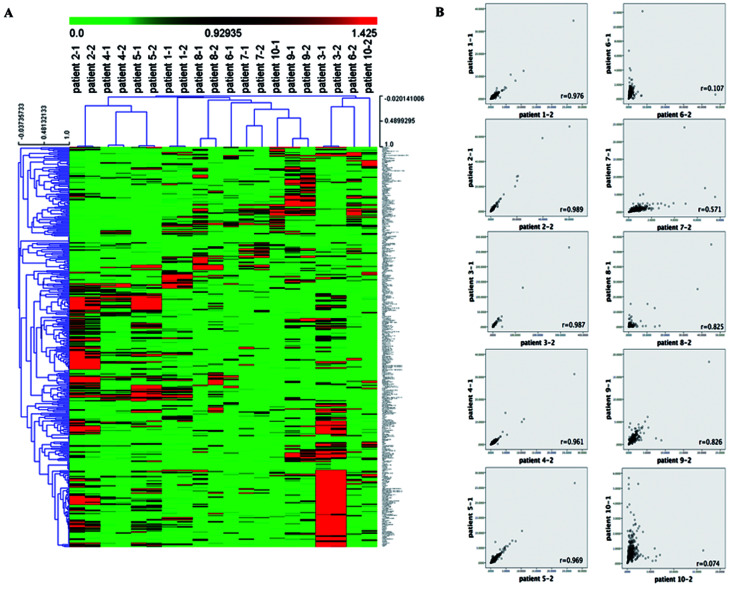
The overall metabolomics profile of RAML patients before and post chemotherapy. (A) Heat map representation of 390 metabolites between patients before chemotherapy (patient 1-1 to patient 10-1) and post treatment (patient 1-2 to patient 10-2) in hierarchical cluster analysis. Each column represents a patient's sample, each row represents a metabolite. The color scale shows the metabolite level in serum, the brighter red color indicates the higher levels; similarly, the brighter green color means the lower content t of the metabolite. (B) The metabolites correlation scatter diagram of the 10 patients before and after chemotherapy. In the plots, each dot is a metabolite, the *y*-axis shows the metabolite level in the blood serum before the chemotherapy, the *x*-axis shows the metabolite level in the blood serum post the chemotherapy. Correlation *r* values are shown on the plots.

We firstly used the unsupervised hierarchical cluster analysis to analyze the 390 metabolites detected in the serum samples from the 10 RAML patients before and post chemotherapy. As shown in Fig. 1A, the samples were clustered based on the metabolites Pearson correlation between samples. Obviously, the metabolomics profile is very heterogenetic in RAML patients, with no common signature among patients. Contrarily, the metabolites in the serum before and post chemotherapy displayed almost identical in each RAML patient except the samples before and post chemotherapy from patients 6 and 10 that did not cluster together. We further analyzed the metabolites in each patient before and post chemotherapy (Fig. 1B), which revealed that the Pearson correlation *r*-values between before and post chemotherapy samples are high in almost all the patients (*r* = 0.728 ± 0.360), except patients 6 (*r* = 0.107) and 10 (*r* = 0.074). Taken together, the RAML patients may have very diverse metabolomics profile. RAML is hematological malignancies with high heterogeneity, and patients were undergoing different chemotherapy regimens. The different chemotherapy regimens may have influence on the metabolites since we found the metabolomics profile was very heterogeneous across the RAML patients, however we cannot exclude the influence of RAML disease heterogeneous on metabolomics. On the other hand, for individual patient, the salvage chemotherapy did not significantly change the metabolomics profile before and the post the chemotherapy, which indicated the different chemotherapy regimens had very limited influence on metabolomics profile for each individual RAML patients. Noteworthily these chemotherapy regimens were all based on cytarabine and served as salvage chemotherapy, and the patients underwent multi-chemotherapies and didn't show response to the last salvage chemotherapy as the disease status did not change upon this one-time chemotherapy. This could be a reason that the last salvage chemotherapy had minimal impact on the metabolomics for individual patient.

**Fig. 2:**
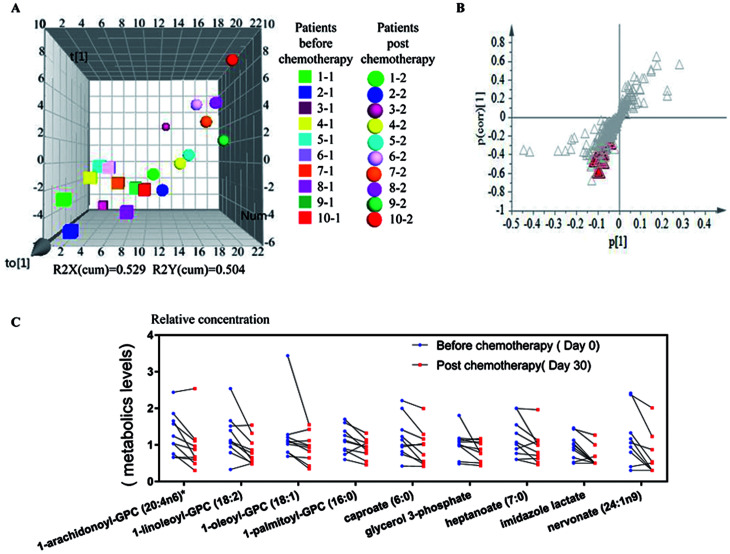
Significantly changed metabolites in blood serum between samples before and post chemotherapy. (A) Score plot from OPLS-DA model showing the discrimination of samples from before and post chemotherapy in 3 dimensions. Square shapes represent samples from patients before treatment, and round shapes represent patient samples after treatment. Samples from each patient were labeled with different colors. (B) *S*-plot derived from OPLS-DA model. Metabolites playing key roles for separation are marked with red triangle. (C) The relative levels of significantly changed 9 metabolites between patient samples before and post chemotherapy, which were all the metabolites levels were normalized to the median level of each compound at the normalization step.

We further employed partial least squares-discriminant analysis (PLS-DA), which is a supervised approach that ranks variables' predictive capacities within a multivariate context, in this case, to identify individual metabolites that are responsible for distinguishing sample differences (Fig. 2A and B). According to the analysis, we identified 9 metabolites from patients' samples before and after chemotherapy as listed in Table 2 and shown in Fig. 2B as red triangles. These 9 metabolites existed in all the 20 samples. The 9 metabolites included 1 amino acid and 8 lipids (Table 2), and it was worth noticing that all these 9 compounds were mostly significantly down regulated after chemotherapy as shown in Fig. 2C. The results indicated that these 9 compounds might be associated with the treatment of refractory leukemia, or play roles in response to the treatments.

**Table 2:** List of the 9 significantly different metabolites before and after chemotherapy

Super pathway	Sub pathway	Biochemical name	Fold change	*p*-value
Amino acid	Histidine metabolism	Imidazole lactate	0.72	0.0071
Lipid	Glycerolipid metabolism	Glycerol 3-phosphate	0.84	0.0495
	Long chain fatty acid	Nervonate (24:1n9)	0.68	0.0161
	Lysolipid	1-Arachidonoyl-GPC (20:4n6)^a^	0.72	0.0076
		1-Linoleoyl-GPC (18:2)	0.74	0.0113
		1-Oleoyl-GPC (18:1)	0.79	0.0249
		1-Palmitoyl-GPC (16:0)	0.84	0.0203
	Medium chain fatty acid	Caproate (6:0)	0.78	0.0142
		Heptanoate (7:0)	0.82	0.0288

a The biochemical name is identified but has not been confirmed based on a standard.

Metabolomics is progressively being used for diagnosing cancer, predicting its recurrence, and determining prognosis which aims to comprehensively assessing endogenous metabolites including amino acids, carbohydrates, lipids, peptides, nucleic acids, and vitamins in specific cells, tissues, or bodily fluids at a given time.^23^ So far, a great number of metabolomic studies focusing on various cancers including AML, CRC, gastric cancer, pancreatic cancer, and liver cancer have been performed for identifying novel cancer biomarkers and developing cancer therapeutics.^15–19^ Generally, there are main analytical platforms for metabolomics analysis: gas chromatography (GC), liquid chromatography (LC), capillary electrophoresis (CE) coupled to MS and NMR spectroscopy. Each has its own unique advantages and disadvantages; for example, NMR is highly selective and non-destructive but with relatively low sensitivity. Musharraf *et al.* recently employed ^1^H NMR spectroscopy to investigate the serum of ALL and AML patients and compared with two controls (healthy and aplastic anemia).^38^ Only thirty-seven putative metabolites were identified using Carr–Purcell–Meiboom–Gill (CPMG) sequence. MS-based metabolomic profiling is sensitive and robust, requiring certain process of sample preparation. Evidences have already demonstrated their (especially GC-MS and LC-MS) potential as powerful analytical method for broad-spectrum identification and quantification of metabolites in cells, tissues, or bodily fluids in human health and disease state.^31,39,40^ In the present study, we took advantage of an established non-targeted metabolomic profiling platform that combined UPLC/MS/MS with GC/MS, a total of 390 named metabolites were identified in the tested 20 samples, which uncovered so far the broadest serum metabolome for acute leukemia patients as compared with previous studies.^38,41–47^ Moreover, those 390 metabolites were mapped to 8 super-pathways and 71 sub-pathways. Obviously, most of the central metabolic pathways were included among the identified metabolites and we believed this non-targeted metabolomic profiling platform successfully elucidated the serum metabolome for RAML patients.

Cancer cells usually exhibits unique metabolic patterns to supports their growth and proliferation.^48^ The most well characterized Warburg effect is observed in many types of cancers, which generally exhibits disturbance in glycolysis. It is characterized by an increase in glucose uptake and lactate production, as well as a decrease in oxidative phosphorylation.^49^ Moreover, increased glutamine metabolism is another commonly observed metabolic alteration, which plays important roles in balancing cellular redox homeostasis and supports the growth and proliferation of cancer cells.^50^ AML is a life threatening hematological disease with diverse genetic abnormalities. Previous metabolomic studies linked AML with perturbation of metabolic pathways included glucose metabolism, fatty acid metabolism, glycerophospholipid metabolism and so on. For example by using ^1^H-NMR spectroscopy in combination with multivariate data analysis, Wang *et al.* analyzed the phenotypic characteristics of serum metabolite composition in a cohort of 183 patients with *de novo* acute myeloid leukemia together with 232 age- and gender-matched healthy controls.^42^ The results showed significant serum metabolomic differences involved in multiple metabolic pathways including glycolysis/gluconeogenesis, tricarboxylic acid (TCA) cycle, biosynthesis of proteins and lipoproteins, metabolisms of fatty acids and cell membrane components especially choline and its phosphorylated derivatives.^42^ Meanwhile, Chen *et al.* identified an altered glucose metabolism signature in AML patients, and more importantly, a panel of 6 metabolite biomarkers involved in glucose metabolism is identified with prognostic value for cytogenetically normal AML.^44^ Additionally, aberrant metabolism pathways including glycolysis, TCA cycle, lipoprotein changes, choline and fatty acid metabolisms were reported in ALL and AML patients by Musharraf *et al.*^41^ In the present study, very subtle metabolic differences including only 9 metabolites were changed in RAML patients with salvage chemotherapy, such as imidazole lactate, glycerol 3-phosphate, three fatty acids, and four lysolipids. Interestingly, levels of all these nine metabolites involved in amino acids and lipids metabolism were low which were mostly in agreement with the observations in the previous study.^38^ It should be pointed out that very few beneficial or even bad effects of the treatment in RAML patients were observed in our study, which to some extent mean that the disease here was on the progress and even worsened. These findings suggested that during the development of AML including the initial and advanced stages of disease, both the metabolism of amino acids and lipids played important roles as energy production.^38^

## Conclusions and prospects

In conclusion, here we employed a non-targeted metabolomics profiling platform that combined UPLC/MS/MS with GC/MS together and for the first time disclosed the feature of metabolomics in 10 RAML patients pre and post chemotherapy. A total of 390 metabolites mapped to 8 super-pathways and 71 sub-pathways were identified. 9 metabolites in the blood serum were found to be changed significantly upon one-time chemotherapy, which might be related to the disease status and development and treatment, and may provide potential biomarkers in the future for RAML. However, there were still several deficiencies in this study: the samples number was small and the therapeutic effects of chemotherapy on these enrolled patients were minimal. In the next steps, these findings on metabolic changes need further investigation, in combination with the genomics, proteomics data for the verification.

## Authors contributions

Authors Zhi Zheng, Jun Rao, and Xing Fan designed the study. Liting Xu, and Wenhao Cui conducted the entire experiment. Pingyi Liu, Zhiqiang Peng, Yayue Zhang, and Xinyi Chen participated in the data collection, analysis and interpretation. Pingyi Liu, Li Hou, Fangfang Tou, Zhi Zheng, Jun Rao, and Xing Fan participated in writing and revising of the manuscript. All authors read and approved the final manuscript.

## Ethics approval and consent to participate

All experiments were performed in accordance with the regulations of the Jiangxi Cancer Hospital Institutional Review Boards. The study protocol was reviewed and approved by the ethics committee of Jiangxi Cancer Hospital. Informed consents were obtained from human participants of this study.

## Conflicts of interest

The authors declare no competing financial interest.

## Supplementary Material

RA-008-C7RA13298K-s001
