# Supplementary material for: Metabolomics analysis of salvage chemotherapy on refractory acute myeloid leukemia patients
Source: RSC Adv. 2018 Apr 18;8(26):14445–53. doi: 10.1039/c7ra13298k (PMC9079900; doi:10.1039/c7ra13298k)
Supplement: RA-008-C7RA13298K-s001 [file RA-008-C7RA13298K-s001.pdf]

Electronic Supplementary Material (ESI) for RSC Advances.
This journal is © The Royal Society of Chemistry 2018

Supplementary Table 1 Treatment outcome of CBC and biochemical examination

	Value (mean ± SD)			P1	P 2
	**Day 0**	**Day 15**	**Day 30**		
WBC (* 10^9/L)	10.198 ± 9.836	4.119 ± 2.657	5.467 ± 4.392	0.048	0.076
HBG (g/L)	80.8 ± 16.8	71.0 ± 13.3	76.7 ± 17.6	0.002	0.172
PLT (* 10^9/L)	93.2 ± 88.3	60.0 ± 53.8	87.1 ± 85.3	0.042	0.234
ALT (U/L)	20.2 ± 8.9	21.5 ± 13.0	23.6 ± 9.5	0.577	0.222
AST (U/L)	20.1 ± 5.4	22.5 ± 11.7	24.6 ± 9.9	0.48	0.154
BUN (mg/dL)	8.53 ± 9.01	7.81 ± 7.65	6.75 ± 4.36	0.809	0.515
Ccr (μmol/L)	79.82 ± 60.50	70.46 ± 38.89	68.73 ± 37.65	0.392	0.259

WBC: white blood cell; HBG: hemoglobin; PLT: platelet; ALT: alanine aminotransferase; AST: aspartate transaminase; BUN: urea nitrogen; Ccr: creatinine

Supplemental Table 2 List of 390 identified metabolites in serum samples

Super Pathway	Sub Pathway	Biochemical Name	KEGG	HMDB
Amino Acid	Glycine, Serine and Threonine Metabolism	glycine	C00037	HMDB00123
		N-acetylglycine		HMDB00532
		sarcosine	C00213	HMDB00271
		dimethylglycine	C01026	HMDB00092
		betaine	C00719	HMDB00043
		serine	C00065	HMDB00187
		N-acetylserine		HMDB02931
		beta-hydroxypyruvate	C00168	HMDB01352
		threonine	C00188	HMDB00167
		N-acetylthreonine		
		homoserine	C00263	HMDB00719
	Alanine and Aspartate Metabolism	alanine	C00041	HMDB00161
		N-acetylalanine	C02847	HMDB00766
		aspartate	C00049	HMDB00191
		asparagine	C00152	HMDB00168
		N-acetylaspartate (NAA)	C01042	HMDB00812
	Glutamate Metabolism	glutamate	C00025	HMDB00148
		glutamine	C00064	HMDB00641
		pyroglutamine*		
	Histidine Metabolism	histidine	C00135	HMDB00177
		1-methylhistidine	C01152	HMDB00001
		trans-urocanate	C00785	HMDB00301
		imidazole lactate	C05568	HMDB02320
	Lysine Metabolism	lysine	C00047	HMDB00182
		N6-acetyllysine	C02727	HMDB00206
		2-aminoadipate	C00956	HMDB00510
		glutarylcarnitine (C5-DC)		HMDB13130
		pipecolate	C00408	HMDB00070
	Phenylalanine and Tyrosine Metabolism	phenylalanine	C00079	HMDB00159
		phenyllactate (PLA)	C05607	HMDB00779
		phenylacetate	C07086	HMDB00209
		4-hydroxyphenylacetate	C00642	HMDB00020
		tyrosine	C00082	HMDB00158
		3-(4-hydroxyphenyl)lactate	C03672	HMDB00755
		phenol sulfate	C02180	HMDB60015
		p-cresol sulfate	C01468	HMDB11635
		3-methoxytyrosine		HMDB01434
		3-(3-hydroxyphenyl)propionate	C11457	HMDB00375
		tyramine O-sulfate		HMDB06409

Tryptophan Metabolism	tryptophan	C00078	HMDB00929
	indolelactate	C02043	HMDB00671
	indoleacetate	C00954	HMDB00197
	indolepropionate		HMDB02302
	3-indoxyl sulfate		HMDB00682
	kynurenine	C00328	HMDB00684
	kynurenate	C01717	HMDB00715
	indoleacetylglutamine		HMDB13240
	tryptophan betaine	C09213	HMDB61115
	C-glycosyltryptophan		
Leucine, Isoleucine and Valine Metabolism	leucine	C00123	HMDB00687
	4-methyl-2-oxopentanoate	C00233	HMDB00695
	isovalerate	C08262	HMDB00718
	isovalerylcarnitine (C5)		HMDB00688
	beta-hydroxyisovalerate		HMDB00754
	beta-hydroxyisovaleroylcarnitine		
	3-methylglutarylcarnitine (C6)		HMDB00552
	alpha-hydroxyisovalerate		HMDB00407
	isoleucine	C00407	HMDB00172
	allo-isoleucine		
	3-methyl-2-oxobutyrate	C00141	HMDB00019
	3-methyl-2-oxovalerate	C00671	HMDB03736
	2-methylbutyrylcarnitine (C5)		HMDB00378
	3-hydroxy-2-ethylpropionate		HMDB00396
	valine	C00183	HMDB00883
	isobutyrylcarnitine (C4)		HMDB00736
	3-hydroxyisobutyrate	C06001	HMDB00336
	alpha-hydroxyisocaproate	C03264	HMDB00746
Methionine, Cysteine, SAM and Taurine Metabolism	methionine	C00073	HMDB00696
	methionine sulfoxide	C02989	HMDB02005
	homocysteine	C00155	HMDB00742
	alpha-ketobutyrate	C00109	HMDB00005
	2-aminobutyrate	C02261	HMDB00650
	2-hydroxybutyrate (AHB)	C05984	HMDB00008
	cysteine	C00097	HMDB00574
	cystine	C00491	HMDB00192
	S-methylcysteine		HMDB02108
	4-amino-2-hydroxybutyrate		

	Urea cycle; Arginine and Proline Metabolism	arginine	C00062	HMDB00517
		urea	C00086	HMDB00294
		ornithine	C00077	HMDB03374
		proline	C00148	HMDB00162
		citrulline	C00327	HMDB00904
		homocitrulline	C02427	HMDB00679
		dimethylarginine (SDMA + ADMA)	C03626	HMDB01539
		N-delta-acetylornithine*		
		trans-4-hydroxyproline	C01157	HMDB00725
		pro-hydroxy-pro		HMDB06695
	Creatine Metabolism	creatine	C00300	HMDB00064
		creatinine	C00791	HMDB00562
	Polyamine Metabolism	acisoga		
		4-acetamidobutanoate	C02946	HMDB03681
	Glutathione Metabolism	cysteine-glutathione disulfide		HMDB00656
		cys-gly, oxidized		
		5-oxoproline	C01879	HMDB00267
	Gamma-glutamyl Amino Acid	gamma-glutamylglutamine	C05283	HMDB11738
		gamma-glutamylisoleucine*		HMDB11170
		gamma-glutamylleucine		HMDB11171
		gamma-glutamylphenylalanine		HMDB00594
		gamma-glutamyltyrosine		HMDB11741
		gamma-glutamylvaline		HMDB11172
	Dipeptide Derivative	N-acetylcarnosine		HMDB12881
Peptide	Polypeptide	bradykinin, hydroxy-pro(3)		HMDB11728
		HXGXA*		
		HWESASXX*		
		XHWESASXXR*		
		HWESASLLR		
	Fibrinogen Cleavage Peptide	ADSGEGDFXAEGGGVR*		
		DSGEGDFXAEGGGVR*		
		ADpSGEGDFXAEGGGVR*		
	Acetylated Peptides	phenylacetylglutamine	C04148	HMDB06344
Carbohydrate	Glycolysis, Gluconeogenesis,	1,5-anhydroglucitol (1,5-AG)	C07326	HMDB02712

	and Pyruvate Metabolism	glucose	C00031	HMDB00122
		1,3-dihydroxyacetone	C00184	HMDB01882
		pyruvate	C00022	HMDB00243
		lactate	C00186	HMDB00190
		glycerate	C00258	HMDB00139
	Pentose Metabolism	ribulose	C00309	HMDB00621
		ribose	C00121	HMDB00283
		ribitol	C00474	HMDB00508
		xylonate	C05411	HMDB60256
		xylose	C00181	HMDB00098
		xylitol	C00379	HMDB02917
		arabinose	C00216	HMDB00646
		threitol	C16884	HMDB04136
		arabitol	C01904	HMDB00568
		fucose	C01018	HMDB00174
	Glycogen Metabolism	maltotriose	C01835	HMDB01262
		maltose	C00208	HMDB00163
		isomaltose	C00252	HMDB02923
	Disaccharides and Oligosaccharides	sucrose	C00089	HMDB00258
	Fructose, Mannose and Galactose Metabolism	fructose	C00095	HMDB00660
		sorbitol	C00794	HMDB00247
		mannose	C00159	HMDB00169
		mannitol	C00392	HMDB00765
		galactitol (dulcitol)	C01697	HMDB00107
	Aminosugar Metabolism	glucuronate	C00191	HMDB00127
		N-acetylneuraminate	C00270	HMDB00230
		erythronate*		HMDB00613
	Advanced Glycation End-product	erythrulose	C02045	HMDB06293
Energy	TCA Cycle	citrate	C00158	HMDB00094
		cis-aconitate	C00417	HMDB00072
		alpha-ketoglutarate	C00026	HMDB00208
		succinylcarnitine (C4-DC)		
		succinate	C00042	HMDB00254
		fumarate	C00122	HMDB00134
		malate	C00149	HMDB00156
	Oxidative Phosphorylation	acetylphosphate	C00227	HMDB01494
		pyrophosphate (PPi)	C00013	HMDB00250
		phosphate	C00009	HMDB01429
Lipid	Medium Chain Fatty Acid	caproate (6:0)	C01585	HMDB00535
		heptanoate (7:0)	C17714	HMDB00666

	caprylate (8:0)	C06423	HMDB00482
	pelargonate (9:0)	C01601	HMDB00847
	caprate (10:0)	C01571	HMDB00511
	10-undecenoate (11:1n1)		
	laurate (12:0)	C02679	HMDB00638
	5-dodecenoate (12:1n7)		HMDB00529
	myristate (14:0)	C06424	HMDB00806
	myristoleate (14:1n5)	C08322	HMDB02000
	pentadecanoate (15:0)	C16537	HMDB00826
	palmitate (16:0)	C00249	HMDB00220
	palmitoleate (16:1n7)	C08362	HMDB03229
	margarate (17:0)		HMDB02259
	10-heptadecenoate (17:1n7)		HMDB60038
Long Chain Fatty Acid	stearate (18:0)	C01530	HMDB00827
	oleate (18:1n9)	C00712	HMDB00207
	cis-vaccenate (18:1n7)	C08367	HMDB03231
	nonadecanoate (19:0)	C16535	HMDB00772
	10-nonadecenoate (19:1n9)		HMDB13622
	arachidate (20:0)	C06425	HMDB02212
	eicosenoate (20:1)		HMDB02231
	erucate (22:1n9)	C08316	HMDB02068
	nervonate (24:1n9)	C08323	HMDB02368
	stearidonate (18:4n3)	C16300	HMDB06547
	eicosapentaenoate (EPA; 20:5n3)	C06428	HMDB01999
	docosapentaenoate (n3 DPA; 22:5n3)	C16513	HMDB01976
	docosahexaenoate (DHA; 22:6n3)	C06429	HMDB02183
	linoleate (18:2n6)	C01595	HMDB00673
Polyunsaturated Fatty Acid (n3 and n6)	linolenate [alpha or gamma; (18:3n3 or 6)]	C06426	HMDB03073
	dihomo-linolenate (20:3n3 or n6)	C03242	HMDB02925
	arachidonate (20:4n6)	C00219	HMDB01043
	adrenate (22:4n6)	C16527	HMDB02226
	docosapentaenoate (n6 DPA; 22:5n6)	C16513	HMDB01976
	docosadienoate (22:2n6)	C16533	HMDB61714
	dihomo-linoleate (20:2n6)	C16525	HMDB05060
	mead acid (20:3n9)		HMDB10378
Fatty Acid,	15-methylpalmitate		

Branched	17-methylstearate		
Fatty Acid, Dicarboxylate	2-hydroxyglutarate	C02630	HMDB00606
	hexadecanedioate	C19615	HMDB00672
	octadecanedioate		HMDB00782
	3-carboxy-4-methyl-5-propyl-2-furanpropanoate (CMPF)		HMDB61112
Fatty Acid, Methyl Ester	palmitate, methyl ester	C16995	
	linoleate, methyl ester		
Fatty Acid, Amide	palmitic amide		
	oleamide	C19670	HMDB02117
Fatty Acid, Amino	2-aminoheptanoate		
	2-aminooctanoate		HMDB00991
Fatty Acid Metabolism (also BCAA Metabolism)	butyrylcarnitine (C4)	C02862	HMDB02013
	propionylcarnitine (C3)	C03017	HMDB00824
Fatty Acid Metabolism(Acyl Carnitine)	acetylcarnitine (C2)	C02571	HMDB00201
	3-hydroxybutyrylcarnitine (1)		HMDB13127
	hexanoylcarnitine (C6)		HMDB00705
	octanoylcarnitine (C8)	C02838	HMDB00791
	decanoylcarnitine (C10)		HMDB00651
	cis-4-decenoylcarnitine (C10:1)		
	laurylcarnitine (C12)		HMDB02250
	palmitoylcarnitine (C16)	C02990	HMDB00222
	stearoylcarnitine (C18)		HMDB00848
	oleoylcarnitine (C18:1)		HMDB05065
Carnitine Metabolism	deoxycarnitine	C01181	HMDB01161
	carnitine	C00318	HMDB00062
Ketone Bodies	acetoacetate	C00164	HMDB00060
	3-hydroxybutyrate (BHBA)	C01089	HMDB00357
Fatty Acid, Monohydroxy	2-hydroxydecanoate		
	2-hydroxypalmitate		HMDB31057
	2-hydroxystearate	C03045	
	3-hydroxyoctanoate		HMDB01954
	3-hydroxydecanoate		HMDB02203
	13-HODE + 9-HODE		
Eicosanoid	leukotriene B4	C02165	HMDB01085
	prostaglandin B2	C05954	HMDB04236
	5-HETE	C04805	HMDB11134
	5-KETE	C14732	HMDB10217

Endocannabinoid	oleoyl ethanolamide		HMDB02088
Inositol Metabolism	myo-inositol	C00137	HMDB00211
	chiro-inositol	C19891	HMDB34220
	inositol 1-phosphate (I1P)	C04006	HMDB00213
Phospholipid Metabolism	choline	C00114	HMDB00097
	glycerophosphorylcholine (GPC)	C00670	HMDB00086
	ethanolamine	C00189	HMDB00149
	phosphoethanolamine	C00346	HMDB00224
Lysolipid	1-palmitoyl-GPC (16:0)		HMDB10382
	2-palmitoyl-GPC (16:0)*		HMDB61702
	1-palmitoleoyl-GPC (16:1)*		HMDB10383
	1-stearoyl-GPC (18:0)		HMDB10384
	2-stearoyl-GPC (18:0)*		
	1-oleoyl-GPC (18:1)		HMDB02815
	1-linoleoyl-GPC (18:2)	C04100	HMDB10386
	1-linolenoyl-GPC (18:3)*		
	1-arachidonoyl-GPC (20:4n6)*	C05208	HMDB10395
	1-palmitoyl-GPE (16:0)		HMDB11503
	1-stearoyl-GPE (18:0)		HMDB11130
	1-oleoyl-GPE (18:1)		HMDB11506
	1-linoleoyl-GPE (18:2)*		HMDB11507
	1-arachidonoyl-GPE (20:4n6)*		HMDB11517
	1-palmitoyl-GPI (16:0)*		HMDB61695
	1-stearoyl-GPI (18:0)		HMDB61696
	1-oleoyl-GPI (18:1)*		
	1-arachidonoyl-GPI (20:4)*		HMDB61690
	1-palmitoyl-GPA (16:0)	C04036	HMDB00327
	1-arachidonoyl-GPA (20:4)		
	1-palmitoyl-GPG (16:0)*		
Lysoplasmalogen	1-(1-enyl-palmitoyl)-GPE (P-16:0)*		
	1-(1-enyl-oleoyl)-GPE (P-18:1)*		
Glycerolipid Metabolism	glycerol	C00116	HMDB00131
	glycerol 3-phosphate	C00093	HMDB00126
Monoacylglycerol	1-palmitoylglycerol (16:0)		HMDB31074
	1-stearoylglycerol (18:0)	D01947	HMDB31075
	1-oleoylglycerol (18:1)		HMDB11567

	2-oleoylglycerol (18:1)		
	1-arachidonylglycerol (20:4)	C13857	HMDB11572
Diacylglycerol	1,2-dipalmitoylglycerol		HMDB07098
Sphingolipid Metabolism	palmitoyl sphingomyelin (d18:1/16:0)		
	stearoyl sphingomyelin (d18:1/18:0)	C00550	HMDB01348
	sphingosine	C00319	HMDB00252
Sterol	lathosterol	C01189	HMDB01170
	cholesterol	C00187	HMDB00067
	7-alpha-hydroxycholesterol	C03594	HMDB01496
	7-beta-hydroxycholesterol		HMDB06119
	7-alpha-hydroxy-3-oxo-4-cholestenoate (7-Hoca)	C17337	HMDB12458
	cholestanol	C12978	HMDB00908
	beta-sitosterol	C01753	HMDB00852
	campesterol	C01789	HMDB02869
Steroid	pregnenolone sulfate		HMDB00774
	21-hydroxypregnenolone disulfate	C05485	HMDB04026
	5alpha-pregnan-3beta,20alpha-diol disulfate		
	pregnen-diol disulfate*		
	pregn steroid monosulfate*		
	cortisol	C00735	HMDB00063
	dehydroisoandrosterone sulfate (DHEA-S)	C04555	HMDB01032
	epiandrosterone sulfate	C07635	HMDB00365
	androsterone sulfate		HMDB02759
	androstenediol (3beta,17beta) disulfate (1)*	C04295	HMDB03818
	androstenediol (3beta,17beta) disulfate (2)*	C04295	HMDB03818
	5alpha-androstan-3beta,17alpha-diol disulfate		
	5alpha-androstan-3alpha,17beta-diol		

		disulfate		
		5alpha-androstan-3beta,17beta-diol disulfate	C12525	HMDB00493
		andro steroid monosulfate 2*	C04555	HMDB02759
	Primary Bile Acid Metabolism	cholate	C00695	HMDB00619
		glycocholate	C01921	HMDB00138
		taurocholate	C05122	HMDB00036
		chenodeoxycholate	C02528	HMDB00518
		glycochenodeoxycholate	C05466	HMDB00637
		taurochenodeoxycholate	C05465	HMDB00951
	Secondary Bile Acid Metabolism	deoxycholate	C04483	HMDB00626
		glycodeoxycholate	C05464	HMDB00631
		glycolithocholate sulfate*	C11301	HMDB02639
		taurolithocholate 3-sulfate	C03642	HMDB02580
		ursodeoxycholate	C07880	HMDB00946
		glycoursodeoxycholate		HMDB00708
		glycocholenate sulfate*		
		taurocholenate sulfate*		
Nucleotide	Purine Metabolism, (Hypo)Xanthine/Inosine containing	inosine	C00294	HMDB00195
		hypoxanthine	C00262	HMDB00157
		xanthine	C00385	HMDB00292
		urate	C00366	HMDB00289
		allantoin	C02350	HMDB00462
	Purine Metabolism, Adenine containing	adenosine	C00212	HMDB00050
		N1-methyladenosine	C02494	HMDB03331
	Purine Metabolism, Guanine containing	guanosine	C00387	HMDB00133
		7-methylguanine	C02242	HMDB00897
		N2,N2-dimethylguanosine		HMDB04824
	Pyrimidine Metabolism, Orotate containing	orotate	C00295	HMDB00226
	Pyrimidine Metabolism, Uracil containing	uridine	C00299	HMDB00296
		pseudouridine	C02067	HMDB00767
		beta-alanine	C00099	HMDB00056
		N-acetyl-beta-alanine	C01073	
	Pyrimidine Metabolism, Thymine	5,6-dihydrothymine	C00906	HMDB00079
		3-aminoisobutyrate	C05145	HMDB03911

	containing			
	Nicotinate and Nicotinamide Metabolism	quinolinate	C03722	HMDB00232
		nicotinamide	C00153	HMDB01406
		trigonelline (N'-methylnicotinate)	C01004	HMDB00875
		N1-Methyl-2-pyridone-5-carboxamide	C05842	HMDB04193
	Pantothenate and CoA Metabolism	pantothenate	C00864	HMDB00210
Cofactors and Vitamins	Ascorbate and Aldarate Metabolism	ascorbate (Vitamin C)	C00072	HMDB00044
		threonate	C01620	HMDB00943
		arabonate	C00878	HMDB00539
		oxalate (ethanedioate)	C00209	HMDB02329
	Tocopherol Metabolism	alpha-tocopherol	C02477	HMDB01893
		beta-tocopherol	C14152	HMDB06335
		delta-tocopherol	C14151	HMDB02902
		gamma-tocopherol	C02483	HMDB01492
		gamma-CEHC		HMDB01931
	Hemoglobin and Porphyrin Metabolism	heme	C00032	HMDB03178
		bilirubin (Z,Z)	C00486	HMDB00054
		bilirubin (E,E)*		
		bilirubin (E,Z or Z,E)*		
		biliverdin	C00500	HMDB01008
		I-urobilinogen	C05790	HMDB04157
		D-urobilin	C05795	HMDB04161
	Vitamin B6 Metabolism	pyridoxate	C00847	HMDB00017
Xenobiotics	Benzoate Metabolism	hippurate	C01586	HMDB00714
		benzoate	C00180	HMDB01870
		4-hydroxybenzoate	C00156	HMDB00500
		catechol sulfate	C00090	HMDB59724
		O-methylcatechol sulfate		
		4-methylcatechol sulfate		
		methyl-4-hydroxybenzoate	D01400	HMDB32572
		4-ethylphenylsulfate	C13637	
		4-vinylphenol sulfate	C05627	HMDB04072
		propyl 4-hydroxybenzoate sulfate		
	Xanthine Metabolism	caffeine	C07481	HMDB01847
		paraxanthine	C13747	HMDB01860
		theobromine	C07480	HMDB02825
		theophylline	C07130	HMDB01889
		1,3-dimethylurate		HMDB01857
	Food	1,6-anhydroglucose		HMDB00640

Component/Plant	2,3-dihydroxyisovalerate	C04039	HMDB12141
	gluconate	C00257	HMDB00625
	N-acetylalliin*		
	ergothioneine	C05570	HMDB03045
	erythritol	C00503	HMDB02994
	piperine	C03882	HMDB29377
	quinate	C00296	HMDB03072
	stachydrine	C10172	HMDB04827
	tartarate	C00898	HMDB00956
	3-(cystein-S-yl)acetaminophen*		
	4-acetaminophen sulfate	C06804	HMDB59911
	4-acetamidophenol	C06804	HMDB01859
	4-acetamidophenylglucuronide		HMDB10316
	furosemide	D00331	HMDB01933
Drug	indomethacin	C01926	HMDB14473
	metformin	C07151	HMDB01921
	metoprolol	D02358	HMDB01932
	metoprolol acid metabolite*		
	ofloxacin	C07321	HMDB01929
	omeprazole	C07324	HMDB01913
	pantoprazole	C11806	HMDB05017
	phenobarbital	C07434	HMDB15305
	1,2-propanediol	C00583	HMDB01881
	O-sulfo-L-tyrosine		
	2-ethylhexanoate		HMDB31230
	2-hydroxyisobutyrate		HMDB00729
	EDTA	C00284	HMDB15109
Chemical	glycerol 2-phosphate	C02979	HMDB02520
	iminodiacetate (IDA)	C19911	HMDB11753
	trizma acetate	C07182	
	N-methylpipecolate		
	4-hydroxychlorothalonil		
